# Supplementary material for: Work-related stress and sleep problems among small-scale miners in Ghana: The role of psychological factors
Source: PLoS One. 2025 Aug 8;20(8):e0324579. doi: 10.1371/journal.pone.0324579 (PMC12333981; doi:10.1371/journal.pone.0324579)
Supplement: Attachment — Submitted filename: Review comments.docx [file pone.0324579.s003.docx]

**Review comments**

**General comments**

I commend the Authors for the good concept of this manuscript and the presentation also. I submit for a minor revision before the manuscript could be accepted for publication. Authors should take note of the following observations.

1. **Journal guidelines.** Authors should take note of Journal guidelines in presenting the abstract and also ensure that references and referencing style is uniform and also conform to Journal guidelines. (See lines 135 and 466).
2. **Study design.** The study should be referred to as a community based cross-sectional study. The ‘quantitative’ should stand for the method of data collection.
3. **Sample size determination**. Authors should justify the use of a default prevalence in determining the minimum sample size for the study. Authors are also commended for the ‘high’ sample size that was utilized for the study.
4. **Sampling technique.** What is the rationale for starting with purposive sampling and then continuing with probability sampling techniques? To this effect, the first stage as presented in the manuscript cannot be referred to as a stage since no probability sampling technique was applied. It is important to know whether the ‘availability of miners as stated in the inclusion criteria is normally distributed’ in the selected communities to warrant the application of probability sampling technique in selecting the respondents. What is the justification for using a sampling interval of five during field visits? Instead of using ‘randomly selected in line 162 it will be better to state that one miner was selected using a simple random sampling technique of balloting. Authors should explain why a cluster sampling technique was not applied in this study.
5. Authors are commended for the good description of the tools used in the study.
6. Taking into account the title of the study and its design, the inclusion of a conceptual framework will be of relevance.
7. The ‘Adjusted’ in line 40 should be deleted.
